# Delayed intestinal obstruction from an unintentionally retained surgical gauze in a 24-year old woman two years after caesarean section: a case report

**DOI:** 10.1186/s13037-023-00371-y

**Published:** 2023-07-21

**Authors:** Samir Ismail Bashir, Yasir Babiker Ali, Elsadig Mohamed Ali, Hiba Awadelkareem Osman Fadl, Abdelrahman Hamza Abdelmoneim Hamza, Mohammed Mahmmoud Fadelallah Eljack

**Affiliations:** 1 Department of Surgery, Faculty of Medicine and Health Sciences, University of Bakht Alruda, Ad Duwaym, Sudan; 2 General Surgery Resident, Medical Specialization Board (SMSB), Khartoum, Sudan; 3 Department of Haematology, Faculty of Medical Laboratory Sciences, Al-Neelain University, Khartoum, Sudan; 4 Haematology First Specialist, Medical Laboratory, Sudanese National Council for Medical & Health Professions (SNCMHP), Khartoum, Sudan; 5 Clinical Immunology Resident, Medical Specialization Board (SMSB), Khartoum, Sudan; 6 Faculty of Medicine, Al-Neelain University, Khartoum, Sudan; 7 Faculty of Medicine, University of Bakht Alruda, Ad Duwaym, Sudan; 8 Community department, University of Bakht Alruda, University of Bakht Alruda, Ad Duwaym, Ad Duwaym, Sudan

**Keywords:** Gossypiboma, Surgical sponges, Intestinal obstruction, Laparotomy, Foreign body

## Abstract

**Background:**

One of the most common surgical emergencies, intestinal obstruction is rarely the result of an inadvertently retained foreign object (also known as a gossypiboma), which may not present symptoms for a lifetime. It also carries additional legal burdens, which may account for the rarity of its reports.

**Case presentation:**

We report a 24-year-old Sudanese female with a history of emergency Caesarean section two years before the admission presented with abdominal distension and absolute constipation, which was diagnosed as intestinal obstruction with a retained gauzed found within the small intestine. Moreover, a review of recent African-reported cases was done to find relatively similar cases.

**Conclusion:**

Adhering to the standard of care in surgical theaters and integrating new methods of prevention like tagged gauze could help to decrease the rate of such cases in the future.

## Introduction

Forgetting a foreign body such as a mass of cotton material, sponges’ gauze, towels, or even instruments is one of the worst misadventures that a surgeon could ever have, which comes with several legal and social consequences [1] [2]. For this condition, the term gossypiboma (derived from Gossypium for “cotton” and Boma for “a place of concealment”) is commonly used [3]. While it is still one of the main causes of intestinal obstruction, exact incidences are unknown (roughly estimated as 1 out of 1000–1500 intra-abdominal operations) [3]. This could be because they are rarely reported in the literature or because sometimes there is not enough documentation in cases that have been diagnosed [4]. As a result, we can assume that the published reports of this technical oversight are only scratch the surface regarding the actual magnitude of this phenomenon [4].

The most common causes are emergency surgery, unplanned changes in operation, high body mass index, hurried sponge counts, long operations, unstable patient conditions, and inexperienced staff numbers [5]. Even though risk can not be totally eliminated, adhering to good intraoperative surgical practices like sponge counting are usually believed to alleviate the problem [5].

While this condition typically presents with nonspecific clinical features, the patient may experience symptoms for the rest of his life. Moreover, it has the potential to cause serious clinical consequences like ileus, abscess, fistula, and bowel necrosis [6]. Rarely, it may transmigrate into the gastrointestinal lumen without any defects, allowing it to exit the body through the intestines, otherwise Laparoscopic or open surgery would be necessary to remove it [7].

Here we present a Sudanese female with a history of emergency Caesarean section presented with intestinal obstruction features, which was found to be due to a gossypiboma formation.

## Case presentation

A 24-year-old female, gravida 2, para 2, who had previously undergone a Caesarean section at a rural hospital two years ago, arrived at the emergency room with a gradual onset of abdominal distension, abdominal pain, and absolute constipation for two days. She also experienced nausea and frequent vomiting of greenish material however, there is no fever, burning urination, or chest pain. There was no history of renal colic or intestinal obstruction.

The patient was vitally stable upon examination. The abdomen was distended with mildly generalized tenderness, but there was no guarding or rigidity. Superficial palpation revealed no abnormalities, but deep palpation revealed a globular mass on the left iliac fossa (pedunculated) with clearly defined edges. On auscultation bowel sounds were present.

**Fig. 1:**
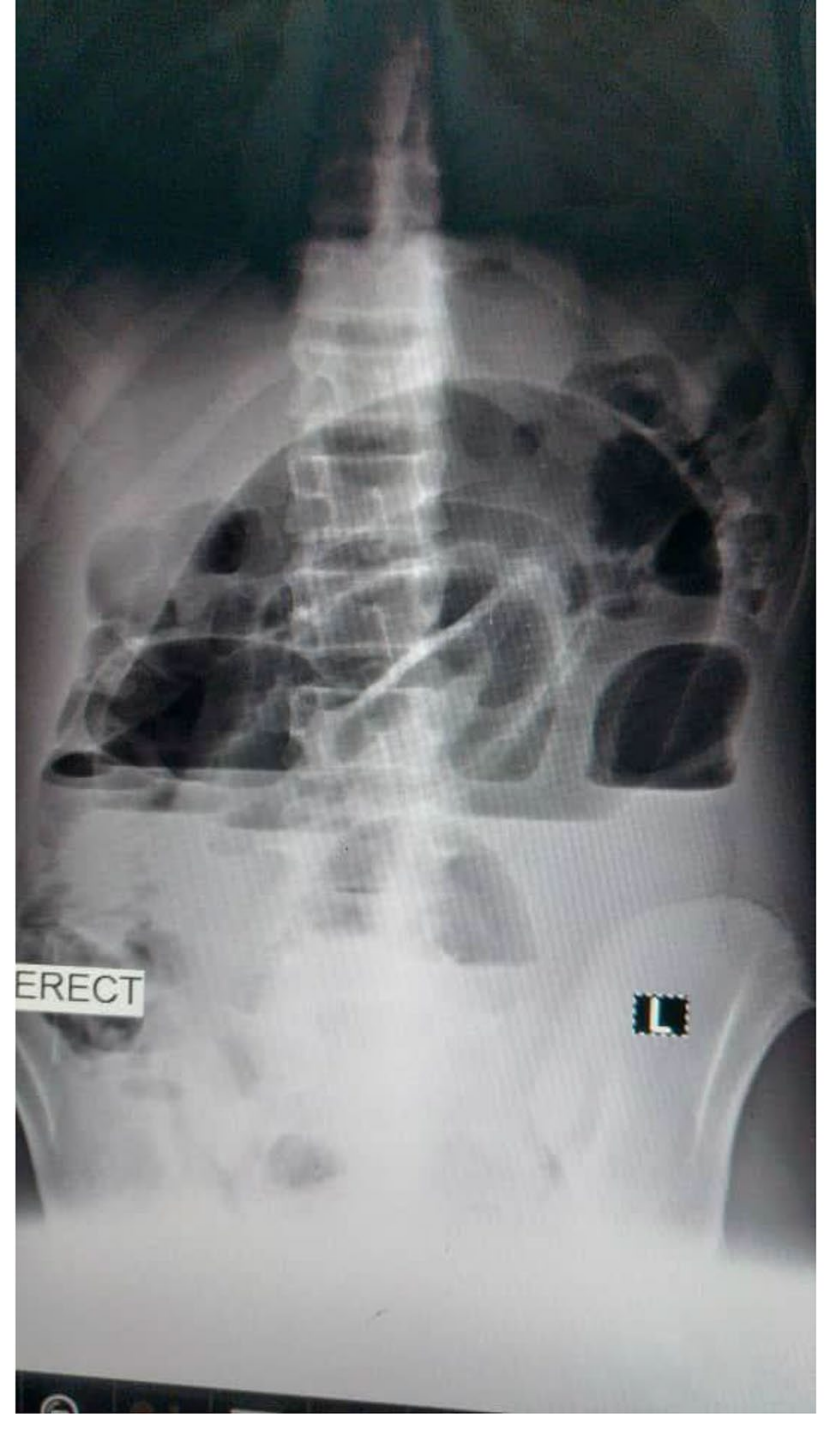
Erect abdominal x-ray revealed multiple air-fluid levels

**Fig. 2:**
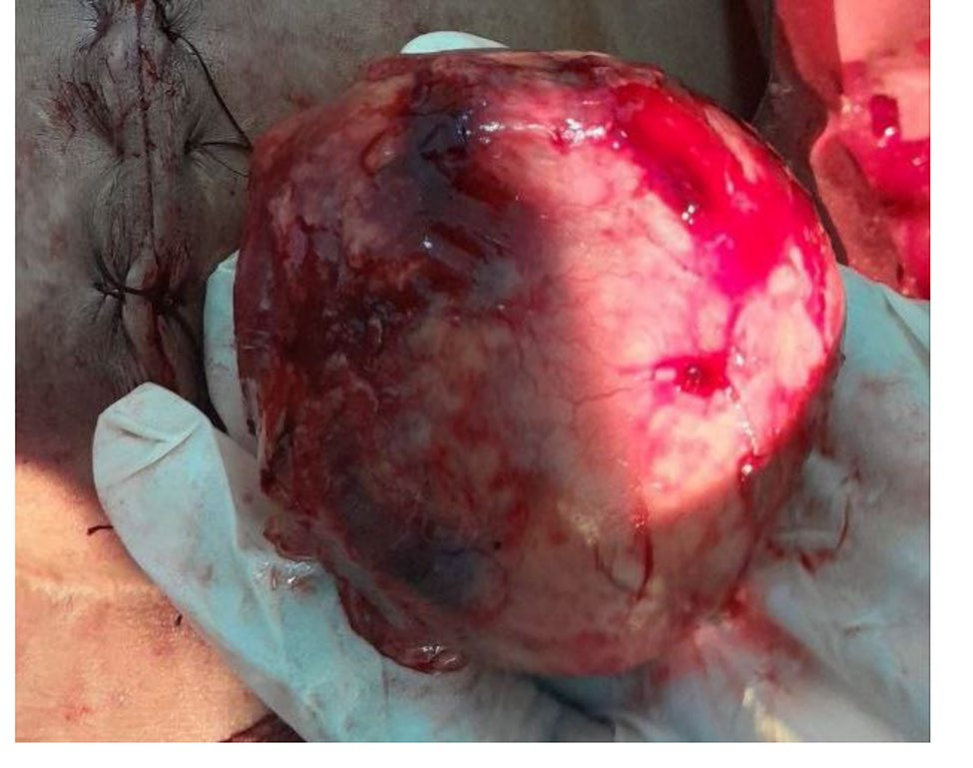
A pink mass attached to the jejunum on two sites causing mechanical intestinal obstruction

TWBCs were 8.1, hemoglobin was 9.8, random blood glucose was 99 mg/dl, platelets were 329, blood urea was 27, serum creatinine 0.7, the c-reactive protein was 27, serum potassium was 3.3 mmol/l, abdominal ultrasound was normal, erect abdominal x-ray revealed multiple air-fluid levels (Fig. 1), while supine revealed a dilated loop of the small intestine. A diagnosis of intestinal obstruction was made, and the patient was optimized before being taken to the theatre. under general anesthesia with tracheal intubation, a midline incision was made with a scalpel, and unipolar diathermy subcutaneous tissue was cut, the muscle was separated, and intraoperative findings showed that the small intestines were found to be adhesively obstructed by a pink mass attached to the jejunum on two closed sites (hanging on it) with serosal attachment, it was firm on consistency measured 10 × 10 cm as illustrated in Fig. 2. The adhesion was released by scissors and the serosal layer was closed by interrupted sutures, then the intestinal and the abdominal cavity was washed, after which, the drain was placed on the pelvic cavity and then the wound closed in layers.

The macroscopic examination revealed a cyst filled with gauze, while the histopathological examination demonstrated gauze material surrounded by fibromuscular connective tissue and mature adipose tissue, with foci of mixed inflammatory cellular infiltration and foreign body giant cell reaction but no malignant features.

The patient received one unit of blood without complications, in the postoperative course the patient was nill per mouth, and receive intravenous fluids and antibiotics for five days. The patient was vitally stable on follow up, with good urine output, and the drain was removed on the fourth day. The patient was discharged on the fifth day and scheduled to see a referring clinic two weeks later.

## Discussion and literature review

Despite being a well-described phenomenon in the surgical world, Gossypiboma is still a frequent problem in multiple countries. This could be partly because not all cases are presented with immediate post-surgical symptoms. Some patients presented after several years due to the delayed non-inflammatory reaction of the body to the retained gauze. Furthermore, although in our case x-rays showed abnormal signs indicating a possible bowel obstruction, regular abdomen X-ray usually doesn’t provide conclusive evidence of the underline existing gossypiboma. That is why CT- abdomen is considered the preferred choice in such cases.

The present study focuses on intestinal obstruction caused by gossypiboma and reported in African countries ten years ago. All cases are females. This may be because they are more exposed to surgical operations due to obstetric surgery.

Several reviews were reported regarding Gossypiboma, one of these reports was written by Wan et al., about retained surgical sponges, which were found in the abdomen in (56%) of the cases. It was commonly detected using computed tomography (61%) and radiography (35%). Furthermore, the median discovery time was of 2.2 years, all of which go in line with the characteristic of our case [8] [13].

Another review was done in Sudan by Suliman et al. and published in 2013, which reviewed several local cases of Gossypiboma in Sudanese females after cesarean section. Which presented with signs of infection and bowel obstruction and required surgical intervention to remove the retained surgical gauze. Luckily all the patients in this report survived until discharge from the hospital [9].

Likewise, Yorke et al. reported a case of intestinal obstruction due to an intraluminal foreign body. In this case, a patient underwent a laparotomy to treat ruptured acute appendicitis. A laparotomy towel was left behind during the operation [10]. In addition, as reported by Emegoakor et al. in Nigeria in 2021 a 28-year-old woman, following an open myomectomy in a private hospital, suffered from colicky abdominal pain, distention, and a mass for 9 months [11]. both patients in this study had similar complications of intestinal obstruction which was the same fate of our patient.

A similar course of events was noticed in the post-myomectomy case reported by Naiem et al. in 2021 which presented with acute abdominal signs after almost 6 months of the primary operation and it was then discovered to be due to infected missed surgical gauze that was then extracted with good patient outcome [12].

For such cases and once the diagnosis has been made, management includes investigations, parenteral antibiotics, intravenous fluids, and emergency laparotomies. In addition, laparoscopic retrieval may also be carried out if the diagnosis is done early [13, 14].

Medical practitioners, especially surgical staff, must be aware of the risk factors that cause retained sponges and take deliberate steps to avoid them [2, 15, 16]. This could involve Keeping a detailed pack and instrument count at the beginning and the end of the surgery, performing a thorough methodical wound examination and abdominal exploration before closure and performing intraoperative radiologic screening or re-exploration when there is a doubt about the accuracy of the final count [17]. A high suspicion index is needed to diagnose gossypiboma and implement prompt interventions to reduce patient morbidity and mortality [16].

One of the promising solutions to decrease the rate of Gossypiboma is the use of tagged surgical gauze which can be detected by regular x-ray. This is expected to decrease the rate of error associated with the use of manual counting (Table 1).

**Table 1:** Summary of intestinal obstruction cases that caused by gossypiboma and reported in African countries ten years ago

Author	Number of cases	Patients ages	Gender	Type of operation	Clinical presentation	Type of post-operative complications	Time to presentation	Possible causes
Suliman et al.2013 (9)	3	Between 24 and 32 ears.	females.	Caesarean section	Abdominal pain, and vomiting	Infections. Small bowel obstruction. Discharged in good condition	5 months to 2 years	NAD
Yorke et al. 0.2019(10)	1	62	female	Appendicitis	Abdominal pain and distension, constipation and vomiting	Infections. bowel obstruction. Discharged in good condition	17 months	NAD
Emegoakor et al. 2021 (11)	1	28	female	myomectomy	Abdominal pain and distension, nausea and occasional diarrhea	Infection, Partial bowel obstruction. Discharged in good condition	9 months	NAD
Naiem e al. 2021 (12)	1	37	female	myomectomy	Abdominal pain, fever, and vomiting	Infections. Bowel obstruction. Discharged in good condition	6 months	NAD
Current case	1	24	female	Caesarean section	Fever, Abdominal pain, distension and constipation	Bowel obstruction. Discharged in good condition	24 months	NAD

## Conclusion

Gossypiboma should be considered even though it is rare in patients with intestinal obstruction, especially if there is a history of abdominal surgery, as it can have serious consequences. To reduce its occurrence and medicolegal ramifications, standard surgical precaution and prevention measures should always be followed, including pre- and post-operative sponge counting, the use of radio-opaque markers, and ongoing staff training.

## Data Availability

The data set used and/or analyzed during the study are available from the corresponding author on reasonable request.
